# Association of circulating Notch1 and VEGF with flow-mediated dilation and aerobic fitness in healthy adults

**DOI:** 10.1515/teb-2025-0006

**Published:** 2025-05-28

**Authors:** Brooke R. Shepley, Nick J. Lester, Lana H. Yacoub, Anthony R. Bain

**Affiliations:** Department of Kinesiology, Faculty of Human Kinetics, 8637 University of Windsor, Windsor, ON, Canada

**Keywords:** Notch1, vascular function, V̇O_2_ peak, vascular endothelial growth factor, flow-mediated dilation, mechanotransduction

## Abstract

Notch1 is a mechanosensitive endothelial receptor that responds to vascular wall shear stress. However, data on Notch1 activity in humans remains limited, particularly regarding its role in endothelial function.

**Objectives:**

Accordingly, the purpose of this study was to determine whether Notch1 is associated with the flow-mediated dilatory (FMD) response and whether it is related to aerobic fitness. A secondary purpose was to determine whether Notch1 is related to concentrations of vascular endothelial growth factor (VEGF).

**Methods:**

Sixteen (8M/8F) young (20–30 yrs old) and healthy (BMI: 25 ± 4.2 kg/m^2^, blood pressure: 117 ± 11.63/69 ± 11.25 mmHg) adults participated in the study. Aerobic fitness was determined by cycle V̇O_2_ peak. An FMD was performed on the brachial artery, and blood samples were taken from an antecubital vein at rest (baseline) and 1 min after cuff deflation (to align with peak vessel dilation). Concentrations of Notch1 extracellular domain (NECD) and VEGF were determined from plasma using enzyme-linked immunosorbent assays.

**Results:**

In contrast to our hypothesis, concentrations of NECD and VEGF did not change throughout the FMD and were unrelated to allometrically scaled FMD values (p all>0.05). Likewise, there was no relationship between changes in NECD and VEGF (p=0.331, r=0.127). However, the change in NECD across the FMD was moderately (r=0.515) and significantly (p=0.024) correlated with V̇O_2_ peak.

**Conclusions:**

These novel data indicate that in healthy young adults, Notch1 activity is linked to aerobic fitness but may not be acutely involved in the shear-mediated vasodilatory response.

## Introduction

Endothelial dysfunction, characterized by impaired endothelium-dependent vasodilation, is a key feature in cardiovascular disease pathogenesis [1]. A contributing factor to endothelial integrity is the friction resulting from blood flowing against the vessel wall, known as shear stress (SS) [2]. Antegrade, or forward-moving, SS is beneficial, strengthening the endothelial barrier and suppressing cell proliferation, while oscillatory SS is damaging, inducing a pro-atherogenic phenotype and impairing endothelial function [2]. As such, understanding the mechanisms of endothelial function associated with altered SS is essential for gaining insight into the risk factors associated with cardiovascular and cerebrovascular diseases – the leading causes of mortality globally [3], [4], [5]. However, the specific pathways of how altered SS impacts endothelial function and how these changes may differ among individuals across fitness levels remain poorly understood.

Emerging evidence suggests that endothelial dysfunction associated with oscillatory SS is largely related to endothelial mechanosensors that detect and respond to mechanical stimuli [6]. A newly described pathway involves the interplay between the mechanical stimulation of SS and the transmembrane receptor, Notch1 [6]. In animal models, Notch1 has been shown to respond explicitly to antegrade SS and linearly correspond to the magnitude of antegrade shear [6], 7]. Indeed, endothelial Notch1 has been shown to exhibit mechanosensory properties in adult arteries necessary for the maintenance of vascular health through improving junctional integrity, cell elongation, and suppression of unorganized endothelial cell proliferation in response to increased antegrade shear [6], 8]. Conversely, suppression of Notch1 signalling is associated with an upregulation of pro-inflammatory molecules, increased cell-to-cell gap junctions, and misaligned endothelial cells [8], 9]. Supporting these findings, atherosclerotic lesions in mice contain reduced expression of Notch1 compared to non-atherosclerotic tissue [6], and inhibition of the Notch1 pathway has been shown to impede vasodilation in response to ischemia in murine endothelial cells [10]. Nevertheless, it is important to acknowledge that vascular endothelial function in humans differs from that observed in mice [11].

Importantly, in canonical Notch1 signalling, antegrade SS stimulates the cleavage of the Notch1 extracellular domain (NECD), which can be quantified as a proxy for Notch1 activity [6]. This pathway is also impacted by circulating vascular endothelial growth factor (VEGF) through upregulating delta-like ligand 4 (Dll4), which binds to Notch1 [12]. Upon activation, Dll4 impedes angiogenesis by suppressing VEGF receptor 2 (VEGFR2) [13]. Interestingly, the binding of VEGF to VEGFR2 is known to stimulate the production of Dll4, which acts as a negative feedback mechanism for angiogenesis [13]. Thus, acute alterations in NECD activity may be associated with a change in VEGF.

Recently, our lab was the first to demonstrate that circulating concentrations of NECD are elevated by ∼50 % in the forearm of healthy adults in response to an acute elevation in antegrade SS following 20 min of forearm cuff occlusion [14]. However, it remains to be determined whether the magnitude of the Notch1 release from antegrade SS is related to the vasodilatory response. Given the increase in NECD during elevated antegrade SS [14], it is plausible that the shear-induced endothelium-mediated vasodilatory response is partly mediated by Notch1. That is, an impaired flow-mediated dilatory (FMD) response, whereby a 1 % decrease in FMD is associated with a 13 % increase in a cardiovascular event [15], may partly be explained by reduced activity of Notch1.

It is well-established that aerobic activity reduces the risk of cardiovascular disease largely through improvements in vascular function [16]. One proposed mechanism for the beneficial impacts of exercise involves exercise-induced increases in antegrade SS that facilitate anti-atherogenic adaptations in the endothelium [17]. Owing to the SS-induced increase in NECD [14], it is reasonable to hypothesize that the considerable increases in SS from exercise may be associated with an upregulation of NECD. Moreover, considering the impact of VEGF on canonical Notch1 signalling, the potential shear-induced changes in NECD may correspond to changes in VEGF. Thus, there is a need to investigate whether Notch1 is altered by chronic exercise training and aerobic fitness, as it may be a prominent contributor to the known beneficial vascular adaptations.

Accordingly, the aims of this study were to 1) determine whether an increase in Notch1 activity is related to the FMD response, 2) determine whether Notch1 activity is associated with VEGF activity during the FMD, and 3) determine whether Notch1 responses to increases in antegrade SS are related to aerobic fitness. It was hypothesized that 1) the change in Notch1 activity would be positively associated with the FMD response, 2) the change in NECD and VEGF would be correlated, and 3) greater aerobic fitness levels would correlate with augmented Notch1 activity.

**Figure 1:**
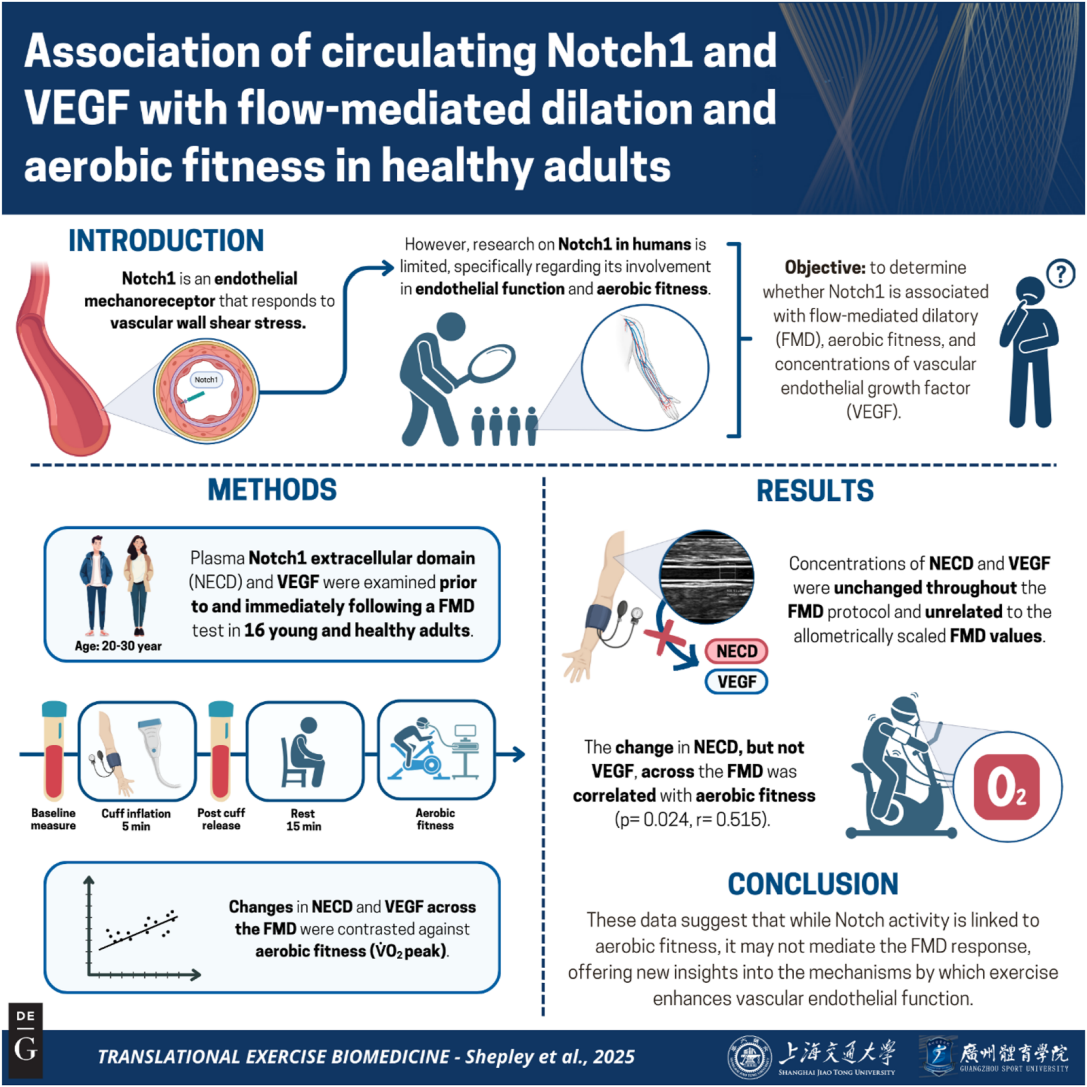
Graphical representation of this study. Key points: (1) this is the first study to examine whether there is an association between circulating Notch1 and vascular endothelial growth factor (VEGF) with the flow-mediated dilatory (FMD) response and aerobic fitness (V̇O_2_ peak) in healthy young adults. (2) Changes in concentrations of Notch1 (extracellular domain) and VEGF were not detectable from resting to the peak shear response of the FMD. (3) There was a positive association between the change in Notch1, but not VEGF, with V̇O_2_ peak. Figure created with BioRender.

The summary of this article is presented in Figure 1.

## Materials and methods

**Figure 2:**
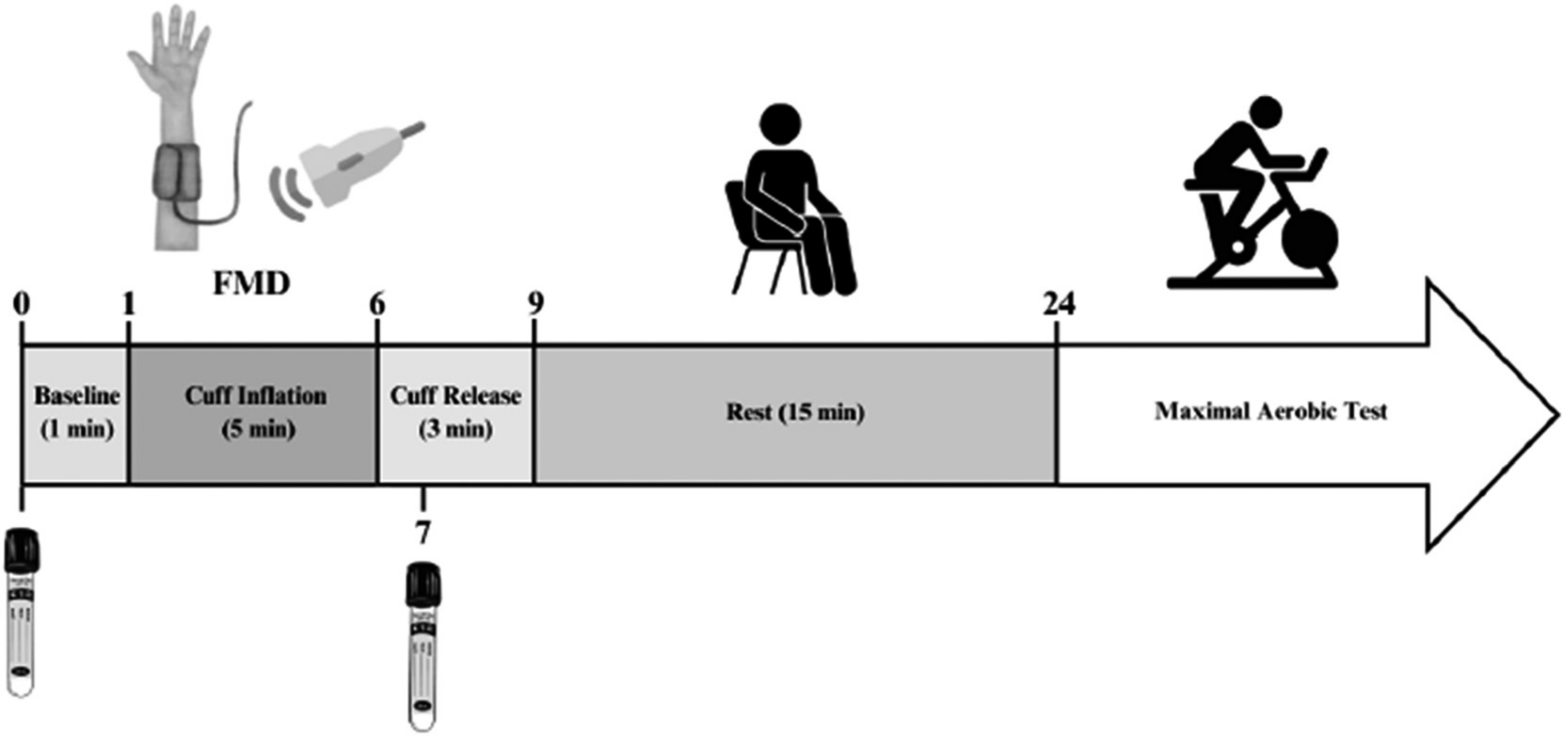
Experimental protocol and timeline, with the numbers indicating time in minutes. The flow-mediated dilation (FMD) protocol included ultrasound recordings during baseline, cuff inflation, and cuff release. The pneumatic cuff was inflated to 220 mmHg to restrict blood flow in the arm. The vacutainer tubes denote when blood samples were taken.

Sixteen (8 females) healthy young adults (20–30 years of age) participated in the study. Participants were free from overt cardiovascular disease, normotensive (117 ± 12/69 ± 11 mmHg), non-obese (25 ± 4 kg/m^2^) and had no history of smoking in the last 12 months. The average V̇O_2_ peak was 40 ± 8 mL/kg/min. The Research Ethics Board at the University of Windsor (REB no. 42638) approved all experimental procedures and protocols in adherence with the principles of the Tri-Council Policy Statement and the *Declaration of Helsinki*, except for registration in a clinical database. Both written and verbal consent were obtained following a briefing of the experimental protocol (Figure 2).

### Experimental design

Participants reported to the lab abstained from alcohol, caffeine, and vigorous exercise 12 h prior to testing, as well as a water-only overnight fast. On the day of the experiment, a venous catheter was inserted into a superficial antecubital vein. A pneumatic cuff was secured around the belly of the forearm on the same arm as the catheter for the FMD. Blood samples were taken prior to cuff inflation (baseline) and 1-min post-cuff deflation (to time align with peak vessel dilation). The FMD followed procedures as outlined in [18] and is outlined below. Following the FMD protocol, participants rested for 15 min and then completed a V̇O_2_ peak ramp test on a cycle ergometer. Figure 2 provides an overview of the experimental timeline.

### Brachial flow-mediated dilation

All procedures were performed in the supine position and followed the guidelines described in Thijssen et al. [18]. Briefly, a narrow pneumatic cuff was positioned around the belly of the forearm. Following 5 min of quiet rest, the brachial artery was imaged using a duplex ultrasound (T3200, Terason, Burlington, MA, USA) for 1 min of baseline measures. Thereafter, the cuff was rapidly inflated to 220 mmHg for 5 min. The cuff was released after 5 min, and ultrasound recordings continued for an additional 3 min. Ultrasound recordings were analyzed offline using custom-designed edge detection software [19]. Shear rate was calculated as 4x the ratio between mean blood velocity (in cm/s) and artery diameter (in cm) [Shear Rate=4 × (mean velocity/diameter)]. To account for differences in baseline brachial diameter, FMD was scaled allometrically [20], and the resulting data was used in all comparative analyses. Allometric scaling was conducted by first determining the natural logarithm of both the baseline and peak diameters (mm). The values obtained were utilized to conduct a regression analysis to find the slope (i.e., β), which was applied to the following formula: Allometrically scaled FMD=peak diameter/(baseline diameter ˆ β) [20].

### Peak exercise test

Aerobic fitness was assessed by using an incremental cycle exercise test on a cycle ergometer (Ergoselect 100, Ergoline). Open-circuit spirometry was used to measure respiratory gases (Quark CPET, COSMED, Chicago, IL, USA). Participants completed either a 20 W, 25 W, 30 W, or 35 W protocol based on their aerobic fitness as determined by the aerobic activity history questionnaire. There was a 2-min warm-up followed by increments of the determined wattage every minute until exhaustion while maintaining 60–80 rotations per minute (RPM). This was followed by a 2-min cool-down (RPM<50). The average test duration was 10.95 ± 1.79 min. Peak exertion was determined when participants met the following criteria: 1) had a respiratory exchange ratio of ≥1.15, 2) failed to maintain ≥60 RPM, and 3) had a plateau in heart rate and V̇O_2_ despite an increased workload.

### Quantification of NECD and VEGF

Whole blood was collected in sodium citrate tubes (Vacutainer, cat. no. 363080, BD Biosciences, San Jose, CA, USA) and centrifuged for 10 min at 1500 g. Plasma was extracted and immediately stored at −80 °C. The plasma samples were then used to determine the concentration of Notch1 ECD using commercially available immunosorbent assays (RayBio, cat. no. ELH-NOTCH1, RayBiotech Life, Peachtree Corners, GA, USA), with an intra- and inter-assay coefficient of variation of <10 % and <12 %, respectively. The detection limit of the assay is 20 pg/mL to 7,000 pg/mL. Samples were read at 450 nm on a microplate reader (BioTek Synergy HT, BioTek Instruments, Winooski, VT, USA) using Gen5 software (Version 1.11, BioTek Instruments). VEGF was quantified using commercially available immunosorbent assays (Bio-Techne, cat. No. DVE00, USA R&D Systems Inc., Minneapolis, MN, USA), with an intra- and inter-assay coefficient of variation of <7 % and <9 %, respectively. The minimal detectable dose is <9.0 pg/mL. Samples were read at 450 nm with a wavelength correction of 540 nm on a microplate reader (BioTek Synergy HT, BioTek Instruments, Winooski, VT, USA) using Gen5 software (Version 1.11, BioTek Instruments).

### Statistical analysis

Data are presented as means ± SD with 95 % confidence intervals (CI), or standard deviation (±SD) for descriptive measures. Tests for significant changes in NECD and VEGF through the FMD protocol were determined by one-tailed paired Student’s *t*-tests (baseline vs. peak dilation). Correlations were determined using one-tailed Pearson’s correlation coefficient (r). Tests for normality were confirmed by Shapiro-Wilk tests. Statistical outliers were identified as values exceeding twice the standard deviation from the mean and were excluded from the analyses where applicable. Following the removal of outliers (either n=1 or n=2, depending on the variable), all values were normally distributed. The removal of outliers did not influence the interpretation of the results; however, the association between V̇O_2_ peak and allometrically scaled FMD no longer reached significance. All statistical analyses were performed in PRISM (GraphPad, Boston, MA, USA). Tests to evaluate sex differences were not performed due to insufficient statistical power, therefore males and females were combined for analysis.

## Results

### Absolute NECD and VEGF concentrations throughout the FMD

**Figure 3:**
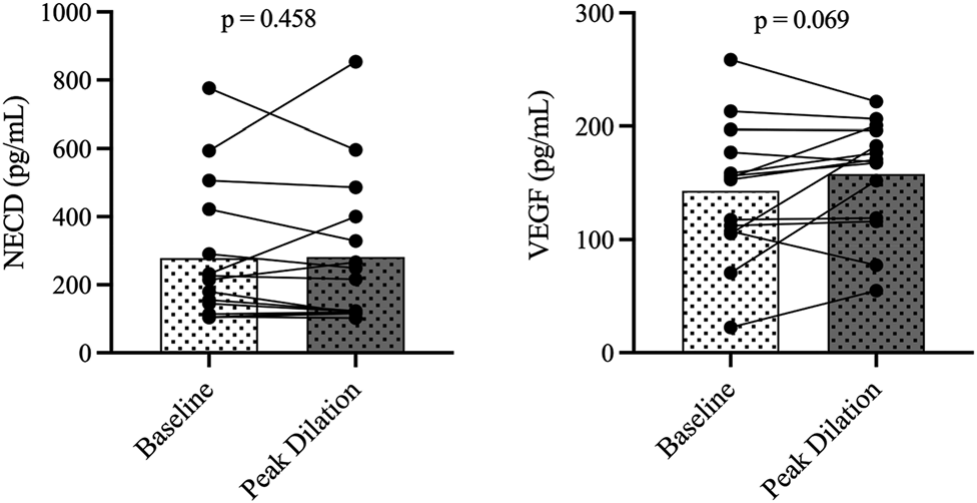
Individual values for NECD (pg/mL) (n=15) (left) and VEGF (pg/mL) (n=14) (right) at baseline and 1 min after cuff release (peak dilation).

The absolute concentrations of NECD and VEGF throughout the FMD protocol are depicted in Figure 3, expressed as pg/mL. *A summary of baseline and peak hemodynamic measures for the FMD test*, *including shear rate and arterial diameter*, *is presented in* Table 1. Compared to baseline, concentrations of NECD were not statistically different following cuff release (peak dilation) (p=0.458). The baseline concentration of NECD was 267.85 ± 202.21 pg/mL with lower and upper 95 % CI of 171.72–363.97 pg/mL. The concentration remained similar following cuff release at 272.08 ± 215.79 pg/mL with lower and upper 95 % CI of 169.51–374.66 pg/mL. Similarly to NECD, baseline VEGF concentrations were unchanged following cuff release (p=0.069). The baseline concentration of VEGF was 145.48 ± 58.72 pg/mL with lower and upper 95 % CI of 117.56–173.39 pg/mL. Following cuff release, the VEGF concentration was 158.79 ± 47.56 pg/mL with lower and upper 95 % CI of 136.19–181.40 pg/mL.

**Table 1:** Baseline and peak hemodynamic values during the flow-mediated dilatory test.

Variable	Mean ± SD
Baseline diameter, mm	3.44 ± 0.79
Peak diameter, mm	3.77 ± 0.87
Diameter change, mm	0.32 ± 0.11
Baseline shear Rate, 1/s	102.36 ± 65.13
Baseline envelope velocity, cm/s	9.01 ± 6.33
Time to peak dilation, sec	52.68 ± 14.21
Response mean shear rate, 1/s	441.52 ± 202.985
Response shear rate AUC +ve	21693.2 ± 7138.17
FMD, %∆	9.45 ± 2.25
Allometrically scaled FMD	1.08 ± 0.02

### Associations of NECD and VEGF with FMD

**Figure 4:**
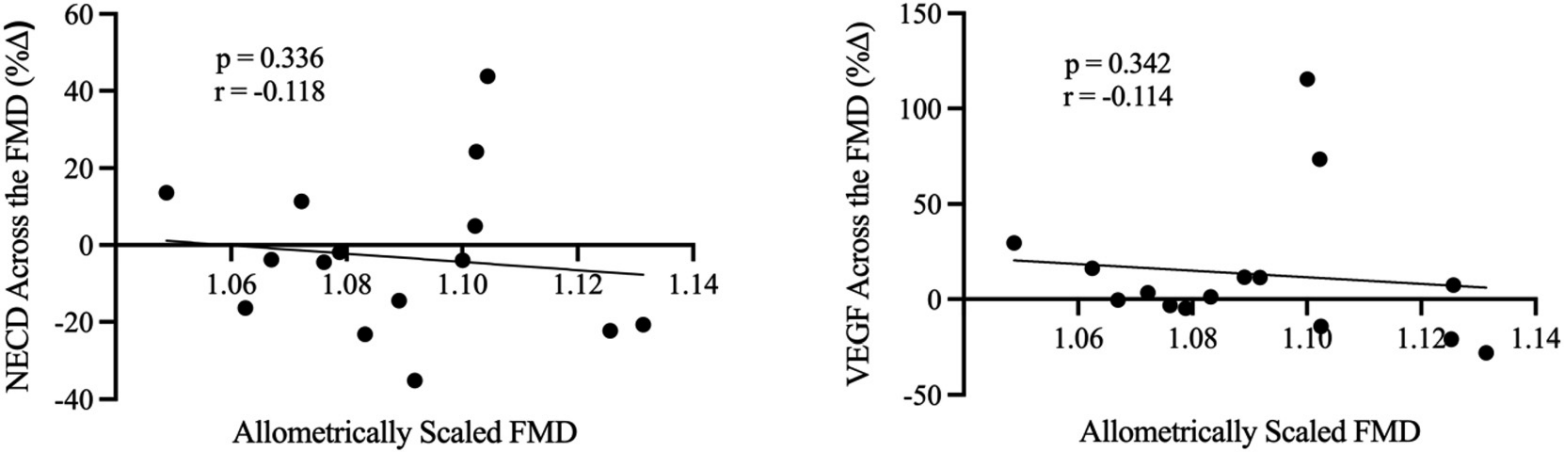
Relationships between allometrically scaled FMD (x-axis) with NECD and VEGF (y-axis) concentrations expressed as percent change across the FMD (n=15). r denotes Pearson’s correlation coefficient (95 % confidence limits).

To investigate the involvement of Notch1 signalling in the FMD response, the percent change of NECD and VEGF concentrations were compared to allometrically scaled FMD, as shown in Figure 4. The percent change of NECD across the FMD was not correlated with allometrically scaled FMD (p=0.336, r= −0.118). Additionally, the change in VEGF across the FMD was also unrelated to allometrically scaled FMD (p=0.342, r= −0.114).

### Relationship between NECD and VEGF

**Figure 5:**
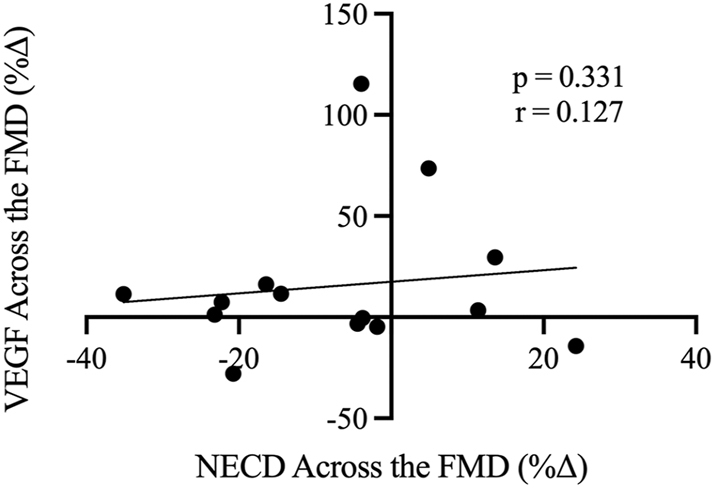
Relationship between the change (%Δ) in NECD across the FMD (x-axis) and the change (%Δ) in VEGF across the FMD (y-axis) (n=14). r denotes Pearson’s correlation coefficient (95 % confidence limits).

To determine the relationship between Notch1 and VEGF activity during the FMD protocol, the percent change in NECD across the FMD was compared to the percent change in VEGF across the FMD, as shown in Figure 5. Contrary to our second hypothesis, the change in NECD was not correlated with the change in VEGF across the FMD (p=0.331, r=0.127). However, NECD and VEGF at baseline were moderately (r= −0.475) and significantly (p=0.042) correlated.

### NECD and VEGF with aerobic fitness

**Figure 6:**
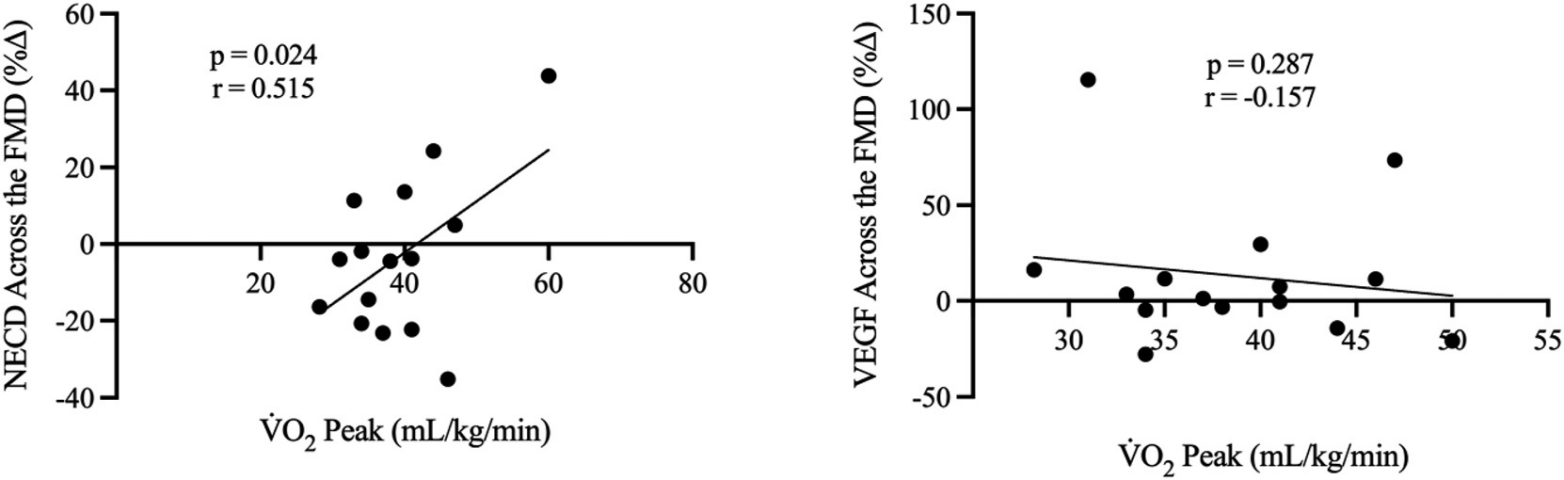
Relationships between V̇O_2_ peak (mL/kg/min) (x-axis) with NECD (n=15) and VEGF (n=15) activity expressed as percent change across the FMD (y-axis). r denotes Pearson’s correlation coefficient (95 % confidence limits).

To determine the relationship between Notch1 and VEGF activity with aerobic fitness, the percent change in NECD and VEGF across the FMD were compared to individual V̇O_2_ peak values, as shown in Figure 6. The change in NECD across the FMD was moderately (r=0.515) and statistically significantly (p=0.024) correlated with V̇O_2_ peak. However, the change in VEGF across the FMD was not correlated to V̇O_2_ peak (p=0.287, r= −0.157). Additionally, there were no significant associations between aerobic fitness and baseline NECD (p=0.287, r= −0.163) or VEGF (p=0.089, r= −0.367). The allometrically scaled FMD was not statistically associated with V̇O_2_ peak (p=0.087. r=0.357).

## Discussion

This is the first study, to our knowledge, to investigate the involvement of Notch1 activity in the FMD response, its relationship with VEGF, and its association with aerobic fitness. The primary findings are that 1) Notch1 activity is not correlated with the FMD response, 2) changes in NECD and VEGF activity during the FMD protocol are unrelated, and 3) the change in NECD concentrations is significantly correlated with V̇O_2_ peak.

### No change in NECD and VEGF across the FMD

Despite our previous report demonstrating increases in circulating NECD following elevated antegrade shear stress [14], there was no change in NECD from baseline to 1-min post-cuff release (Figure 3). In other words, NECD did not change from 5 min of reduced antegrade shear (cuff inflation) and 1 min of increased antegrade shear (cuff deflation). It was surprising that NECD was not elevated during the FMD protocol, considering the interactions between Notch1 and the potent vasodilator, nitric oxide [21], which is largely responsible for the FMD response [22]. Moreover, we have previously reported an elevation in Notch1 with an acute increase in shear stress [14]. However, compared to Badour et al., the lack of NECD change in the present study is likely explained by the differing experimental protocol [14]. That is, in Badour et al. [14], the ischemic condition lasted for 20 min (as opposed to 5 min), and an upper cuff was also placed over the arm (just below the axilla), which was inflated to 40 mmHg to ‘trap’ circulating proteins released from the shear stimulus. In the current study, a proximal pressure cuff to trap circulating NECD was not employed, as it would have interfered with the standardized FMD protocol. A large concentration of NECD released from the FMD may have, therefore, been washed away in the circulation, rendering it relatively undetectable. The 20-min vs. 5-min ischemia may also explain the differences with Badour et al., whereby more prolonged tissue hypoxia may have potentiated NECD release [14].

Unaltered concentrations of VEGF across the FMD protocol may also be due to the duration of blood flow occlusion, as hypoxia is a chief regulator of VEGF release [23] and, therefore, may also require prolonged occlusion to stimulate an upregulation. Moreover, VEGF has been shown to exhibit a delayed release [24], 25], suggesting that a subsequent blood draw at 12–24 h may be necessary for the detection of any alterations in VEGF.

### Interaction between NECD and VEGF

It was hypothesized that the FMD process would stimulate angiogenic activity, followed by downstream Dll4-Notch1 signalling [26], demonstrated by increased VEGF and NECD activity. Nevertheless, despite no significant increases in NECD or VEGF across the FMD and the aforementioned delayed release of VEGF, potential acute relations between NECD and VEGF were still explored to possibly shed light on the *in vitro* data demonstrating mechanistic interactions [13], 23], 26]. Indeed, VEGF and Notch1 function in a competitive manner to regulate angiogenesis, wherein increased levels of VEGF lead to an upregulation of Dll4, subsequently enhancing Notch1 signalling, which in turn inhibits the expression of VEGFR [9], 12], 27], 28]. This was demonstrated by the inverse correlation between NECD and VEGF at baseline (p=0.042), whereby increased NECD corresponds to reduced concentrations of VEGF. However, the individual change in NECD was not related to the change in VEGF across the FMD (Figure 5). These data indicate there are no interactions between the changes in VEGF and NECD acutely following 5 min of blood flow occlusion, at least that can be gleaned from the circulation, or that this stimulus was not robust or long enough to elicit any measurable effects.

### Positive association between V̇O_2_ peak and change in NECD, but not VEGF, across the FMD

Despite the lack of a consistent directional change in NECD and VEGF across the FMD, there was a significant correlation between the V̇O_2_ peak and the change in NECD across the FMD (Figure 6; p=0.024, r=0.515). That is, those with the greatest aerobic fitness, on average, had the greatest release of NECD during the FMD. The correlation between NECD and V̇O_2_ peak indicates that the vascular benefits of aerobic fitness [16], 29] may be partly attributed to Notch1 signalling, whereby Notch1, in non-tumorigenic states, is linked to vascular endothelial protection [6]. Specifically, the Notch1 signalling pathway has been shown to enhance the endothelial barrier by improving junctional integrity, consequently reducing vascular permeability [9], 27], 30], 31]. Indeed, diminished Notch1 expression has deleterious repercussions for the endothelium, including increasing pro-atherogenic signalling, enhanced endothelial adhesion of inflammatory molecules, increased endothelial cell proliferation, and reduced endothelial junctional integrity [6], 8].

**Figure 7:**
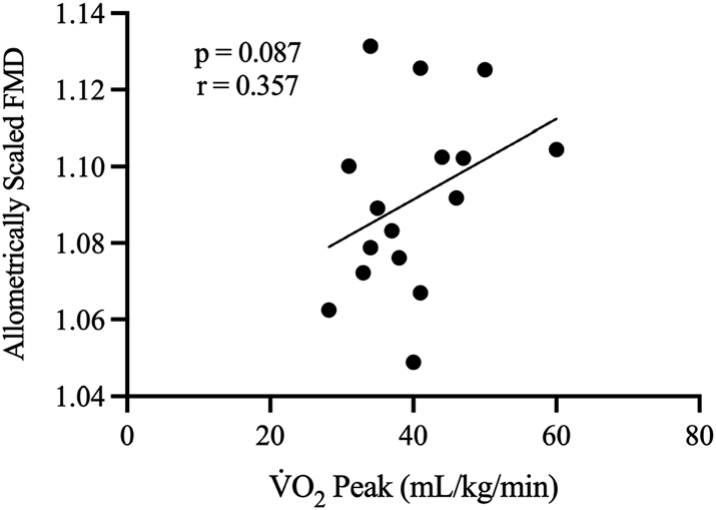
Relationship between V̇O_2_ peak (mL/kg/min) (x-axis) and allometrically scaled FMD (y-axis) (n=16). r denotes Pearson’s correlation coefficient (95 % confidence limits).

Allometrically scaled FMD values were not associated with V̇O_2_ peak (Figure 7), which is consistent with previous studies [32], 33], albeit not a universal finding [34], 35]. However, it is interesting that the change in Notch1 across the FMD was associated with V̇O_2_ peak. These data may point to other vascular protective effects of Notch1 that may be upregulated with improved aerobic fitness, i.e., atheroprotection [10], that are, at least acutely, unrelated to vascular dilation in response to shear stress. Future investigations relating to the relationship between NECD responses to shear stress and endothelial plaque (e.g., across aging and fitness) may provide insight into the observations from this study.

### Considerations

In this initial pilot study (n=16) investigating Notch1 with vascular function and aerobic fitness, we examined NECD and VEGF in the venous circulation while assessing vascular function in the brachial artery. It is also important to note that NECD is an indirect measure of intracellular Notch1 activity. During canonical Notch1 signalling, NECD is cleaved via γ-secretase, resulting in its subsequent release into the bloodstream, enabling convenient quantification of plasma NECD [10]. NICD is then cleaved and translocated to the nucleus, where it regulates atheroprotective gene transcription [13]. As such, NICD would be a more accurate indicator of Notch1 signalling. However, quantification of NICD is not feasible *in vivo* in humans, and NECD as a proxy for Notch1 activity is the best alternative. Additionally, it is important to note that while the typical peak dilation occurs around 60 s, this can vary among participants [22]. Therefore, the ‘peak dilation’ blood draw may not align perfectly with the individual peak dilation times, and the venous vs. arterial compartments must be considered.

Secondly, we did not control for menstrual phases in female participants or contraceptive use, and we were underpowered to perform a sex difference analysis. The different phases of the menstrual cycle may have contributed to a larger variability in the data and should be considered in future studies. Lastly, the participants were young, healthy adults, and therefore, further research is needed to elucidate the implications of Notch1 and aerobic fitness in aging vasculature.

## Conclusions

In summary, our findings suggest that Notch1 signalling in response to increased shear stress is not implicated in endothelial-dependent flow-mediated dilation or related to VEGF. However, this study is the first to demonstrate a positive correlation between Notch1 and aerobic fitness, offering new insights into the mechanistic pathways through which exercise, and consequently aerobic fitness, enhances vascular endothelial function. As Notch1’s function as a vascular endothelial mechanosensor has only been recently described [6], 14], understanding its role in maintaining vascular endothelial health is crucial. To date, most of the literature on Notch1 has focused on its pathogenic role in tumour angiogenesis [26], 36], demonstrating the need for research investigating the role of Notch1 in healthy individuals.
